# Monocyte Titin Gene Expression as a Biomarker of Left Ventricular Dysfunction in Acute Myocarditis

**DOI:** 10.3390/genes17030268

**Published:** 2026-02-26

**Authors:** Spyridon Maragkoudakis, Aleksi Sallo, Ioanna Kontaraki, Emmanouil Marakas Sideras, Gabriela Lilikaki, Onoufrios Malikidis, Konstantinos Fragkiadakis, Eleutherios Kallergis, Nick Kopidakis, Ioannis Kopidakis, Evangelos Zacharis, Vasiliki Katsi, Emmanouil Kampanieris, George Kochiadakis, Emmanouil Simantirakis, Maria Marketou

**Affiliations:** 1 School of Medicine, University of Crete, 71110 Heraklion, Greece; smaragoudakis79@hotmail.com (S.M.); salloalex309@gmail.com (A.S.); kontarai@uoc.gr (I.K.); manolis.1994@windowslive.com (E.M.S.); onoufriosmalikides@gmail.com (O.M.); fragkiadakisk@hotmail.com (K.F.); ekallergis@med.uoc.gr (E.K.); kopidakisg@gmail.com (I.K.); ezacharis@yahoo.gr (E.Z.); manoskampanieris.94@gmail.com (E.K.); kochiadg@gmail.com (G.K.); esimant@uoc.gr (E.S.); 2 Chania General Hospital, 73300 Chania, Greece; gabrielalilikaki@gmail.com; 3 Ippokration General Hospital, 11527 Athens, Greece; vkkatsi@yahoo.gr

**Keywords:** myocarditis, titin, global longitudinal strain

## Abstract

Background: Titin (TTN), a giant structural and signaling protein of striated muscle, participates in intracellular signaling networks and cytoskeletal organization, potentially influencing cell activation, trafficking, and interactions with other tissues, including the heart. Methods: In this pilot study, 29 patients with acute myocarditis and 10 healthy individuals were prospectively enrolled. Peripheral blood was obtained on the first day of hospital admission, total RNA was isolated from peripheral blood mononuclear cells (PBMCs), and TTN mRNA expression was quantified. Results: TTN expression in PBMCs was significantly higher in patients with acute myocarditis compared with healthy controls (*p* = 0.015), corresponding to a 2.8-fold median increase. Moreover, TTN expression showed a strong positive correlation with global longitudinal strain impairment (Spearman’s r = 0.576, *p* < 0.001), a moderate positive correlation with peak hs-cTnI levels (r = 0.435, *p* = 0.021; and a moderate inverse correlation with baseline LVEF (r = −0.421, *p* = 0.025). Conclusions: These findings support a pathophysiological link between TTN-related pathways in peripheral immune cells and myocardial injury in acute myocarditis and raise the hypothesis that TTN expression in PBMCs may serve as a novel biomarker of disease severity and long-term ventricular remodeling. Further studies in larger cohorts are warranted to validate these results and to elucidate the mechanistic role of titin in immune–cardiac cross-talk.

## 1. Introduction

Myocarditis encompasses a spectrum of inflammatory cardiomyopathies triggered by infectious agents, autoimmune processes, toxins, or idiopathic factors, manifest ing clinically from subclinical disease to fulminant heart failure and progression to dilated cardiomyopathy (DCM) [1,2,3]. Although high-sensitivity troponins (hs-cTn) and advances in cardiovascular imaging have improved diagnosis, critical gaps persist in forecasting clinical trajectories, tailoring therapeutic interventions, and deciphering underlying pathophysiological mechanisms [4]. A fundamental clinical challenge in myocarditis is explaining why only a subset of individuals exposed to common triggers—predominantly viral infections—develop clinically significant myocardial inflammation, exhibiting highly variable phenotypes, from spontaneous resolution to fulminant heart failure or chronic dilated cardiomyopathy. Host genetic susceptibility has emerged as the primary determinant, orchestrating interindividual differences in immune activation, viral clearance, and myocardial resilience to inflammatory injury [5,6,7].

Genome-wide association studies have identified immune loci (e.g., HLA-DR4) linked to excessive cytokine production (IL-1β, IL-17, TNF-α), matrix metalloproteinase dysregulation, and fulminant phenotypes [6]. Concurrently, cardiomyopathy-associated variants—particularly in TTN, desmoplakin, and lamin are enriched in myocarditis cohorts (8–30% prevalence vs. <1% controls), supporting a “two-hit” hypothesis: genetic predisposition (hit 1) combined with an environmental insult (hit 2) [8]. TTN, encoding the largest human protein (~3.7 MDa), forms the sarcomeric spring structure, regulating diastolic stiffness, mechanosensing, and contractile force via phosphorylation sites and kinase domains. TTN truncating variants (TTNtv) cause approximately 25% of familial DCM through haploinsufficiency, rendering myocardium vulnerable to stretch-induced remodeling. In myocarditis, TTNtv (8–30% of cases) sensitize cardiomyocytes to viral proteases, amplifying sarcomeric disruption, viral replication, and remodeling toward DCM [2,9]. Beyond germline variants, transcriptomic dysregulation in peripheral blood mononuclear cells (PBMCs) may mirror myocardial pathology [10]. Monocytes, key infiltrators of inflamed myocardium, promote fibrosis via TGF-β signaling and matrix metalloproteinase activity; their mechanosensitive proteome may reflect systemic dysregulation [11,12]. TTN non-cardiomyocyte expression—including endothelial cells, fibroblasts, immune lineages—suggests important roles in cytoskeletal remodeling and signaling beyond the sarcomere [13,14]. In monocytes, TTN may mediate stiffness-sensing, migration, and cytokine secretion, thereby linking peripheral immune activation to cardiac injury [13,15].

No prior study has quantified monocyte TTN mRNA in myocarditis or correlated it with subclinical systolic metrics such as global longitudinal strain (GLS), which is superior to ejection fraction for early detection of systolic dysfunction. This pilot study aims to evaluate TTN mRNA expression in PBMCs as a biomarker of left ventricular systolic dysfunction in acute myocarditis, investigating whether baseline monocyte TTN overexpression predicts subclinical impairment at 1-year follow-up.

## 2. Materials and Methods

### 2.1. Study Population

This two-center prospective pilot study consecutively screened patients admitted with suspected acute myocarditis to the Cardiology departments of the University General Hospital of Heraklion and the collaborating center of the General Hospital of Chania from January 2023 to December 2025. The diagnosis was established on the basis of recent symptom onset (<10 days), clinical presentation, elevated hs-cTnI, electrocardiographic (ECG) findings, and cardiac magnetic resonance imaging (cMRI) [16]. The diagnosis required clinical suspicion plus at least one confirmatory test. More specifically, clinical suspicion was triggered by acute chest pain with hs-cTnI elevation and new ST-T changes, new onset heart failure of unknown origin, unexplained ventricular arrhythmias or conduction abnormalities, or post-viral syndrome with cardiac symptoms. Confirmatory tests included cMRI using the 2018 Modified Lake Louise Criteria, whereby a definite cMRI diagnosis required at least two of the following three findings: regional or global T2 signal increase indicating myocardial edema, early gadolinium enhancement reflecting hyperemia or capillary leak, and subepicardial/midwall late gadolinium enhancement [17]. Endomyocardial biopsy was performed when clinically needed. Supportive criteria included ECG findings such as new left bundle branch block, atrioventricular block, low voltage, or ventricular ectopy; elevated troponins levels; elevated BNP/nt-proBNP; and echocardiographic regional wall motion abnormalities or reduced GLS.

Consecutive patients aged >18 years old presenting with a preceding febrile infectious episode and elevated hs-cTnI levels were included. Symptom onset required at least one of the following: chest pain, dyspnea, new/worsening heart failure, sustained ventricular arrhythmias, or syncope. Diagnosis was confirmed through systematic review of comprehensive clinical histories, laboratory results, inflammatory markers (C-reactive protein, erythrocyte sedimentation rate), and echocardiographic findings in each case.

Control subjects consisted of healthy volunteers matched for age and sex with the myocarditis cohort, who presented to the emergency department with atypical symptoms—such as nausea, dizziness, nonspecific chest discomfort, or upper back pain—and were subsequently confirmed to have a normal cardiovascular evaluation. A detailed history, along with clinical, laboratory and echo data, was thoroughly analyzed, and diagnostic appropriateness was reviewed in every case [16].

We excluded patients with coronary artery disease, myositis, or renal failure. Additional exclusion criteria included previously diagnosed cardiomyopathy or a family history of cardiomyopathy; use of potentially cardiotoxic drugs; autoimmune disease; cardiogenic shock, congenital heart disease, dilated or hypertrophic cardiomyopathy, and arrhythmias not related to myocarditis; other immune diseases; and treatment with glucocorticoids or immunosuppressants prior to specimen collection. In all patients, obstructive coronary artery disease was excluded through coronary angiography, either by cardiac catheterization or by computed tomography in cases with very low pretest probability.

This study was conducted in accordance with the principles of the Declaration of Helsinki (1975). The study protocol was approved by the hospital’s Scientific and Ethics Committee. Written informed consent was obtained from all participants prior to enrollment.

### 2.2. Echocardiography

All participants underwent a standard M-mode and two-dimensional (2D) echocardiographic examination, in accordance with the recommendations of the American Society of Echocardiography and the European Association of Echocardiography [18]. In patients with acute myocarditis, echocardiographic assessment was performed within the first 48 h of hospital admission and at one-year follow-up. Two-dimensional speckle-tracking strain analysis was conducted on grayscale images of the left ventricle using EchoPAC software (GE Medical Systems, Chicago, IL, USA), and peak GLS was quantified.

For strain analysis, the endocardial border was manually delineated at end-systole, and the region of interest was adjusted to encompass the full myocardial wall thickness. The software automatically tracked myocardial segments, accepting those with adequate tracking quality and excluding poorly tracked segments; however, manual adjustments were permitted based on visual assessment by the operator.

All GLS measurements were independently performed by two experienced investigators who were blinded to clinical data. Intra-observer variability for GLS assessment was less than 5%.

### 2.3. cMRI

All patients with confirmed myocarditis underwent cMRI with LGE protocols within the first week of hospital admission and at one-year follow-up to assess myocardial inflammation, edema, and scar formation. cMRI examinations were performed in accordance with the Lake Louise criteria established by the International Consensus Group on cMRI Diagnosis of Myocarditis. LGE were acquired after intravenous administration of gadolinium diethylenetriaminepentaacetate [19].

### 2.4. RNA Isolation and Quantitative Real-Time PCR

Peripheral blood mononuclear cells (PBMCs) were isolated by gradient centrifugation using Lymphoprep (Stem Cell Technologies Inc., Vancouver, BC, Canada). Total RNA was isolated using TRI-Reagent (Sigma-Aldrich Co. LLC, Burlington, MA, USA) according to the manufacturer’s instructions. RNA concentration and purity were assessed spectrophotometrically.

Complementary DNA (cDNA) was synthesized from total RNA using the FastGene Scriptase II cDNA 5× ReadyMix (LS65; Nippon Genetics, Duren, Germany), which includes both oligo(dT) and random hexamer primers, according to the manufacturer’s protocol. Quantitative real-time polymerase chain reaction (qPCR) was performed using the Corbett Rotor-Gene 6000 Real-Time PCR detection system and the KAPA SYBR FAST qPCR Kit (Kapa Biosystems, Woburn, MA, USA).

Gene-specific primers were designed to amplify an exon–exon spanning region of the *TTN* gene. GAPDH was used as an internal housekeeping gene for normalization of gene expression levels. All samples were run in duplicate.

Relative gene expression levels were calculated using the comparative Ct (ΔΔCt) method. Amplification specificity was confirmed by melting curve analysis.

### 2.5. Statistical Analysis

Continuous variables are presented as mean ± standard deviation (SD) when approximately normally distributed and as median (interquartile range) when non-normally distributed. Between-group comparisons (myocarditis vs controls) were performed using independent-samples *t*-tests for parametric variables and the Mann–Whitney U test for non-parametric variables. Changes in left ventricular ejection fraction (ΔLVEF) and ΔGLS were calculated as derived variables by subtracting baseline values from follow-up measurements (Δ = follow-up − baseline).

Associations between TTN expression levels and biochemical or echocardiographic parameters were evaluated using correlation analyses. Pearson’s correlation coefficient was used for parametric variables, whereas Spearman’s rank correlation coefficient was applied for non-parametric variables. All tests were two-sided, and a *p* value <0.05 was considered statistically significant. To assess whether TTN mRNA expression provides information beyond myocardial injury, multivariable linear regression analyses were performed. TTN mRNA expression and high-sensitivity hsTnI were entered simultaneously as independent variables. Separate models were constructed with baseline left ventricular ejection fraction and changes ΔLVEF as dependent variables. All statistical tests were two-sided, and a *p* value < 0.05 was considered statistically significant. Statistical analyses were performed using IBM SPSS Statistics software 26.0.

## 3. Results

This prospective pilot study enrolled 29 patients with acute myocarditis (27 males [93%] and 2 females; mean age 32 ± 7 years) and 12 age- and sex-matched healthy controls (4 males and 8 females; mean age 33 ± 5 years). The baseline clinical characteristics are summarized in Table 1. All 29 enrolled patients (100%) completed the 1-year echocardiographic and cMRI follow-up; thus, no patients were lost to follow-up in the longitudinal analyses. Among myocarditis patients, diffuse ST-segment elevations were observed in 62% (18/29) on electrocardiography. Invasive coronary angiography was performed in three patients with a higher pretest probability of coronary artery disease and excluded obstructive disease in all cases. On cMRI, subepicardial LGE consistent with myocarditis was detected in 23 patients (79%), predominantly affecting myocardial segments corresponding to echocardiographic wall-motion abnormalities.

As shown in Table 1, myocarditis patients exhibited a pronounced systemic inflammatory response, with significantly elevated C-reactive protein and white blood cell counts compared with controls (both *p* < 0.01), accompanied by markedly increased high-sensitivity hs-cTnI levels. Baseline systolic function was significantly impaired, with both LVEF and GLS substantially reduced relative to controls.

**Figure 1:**
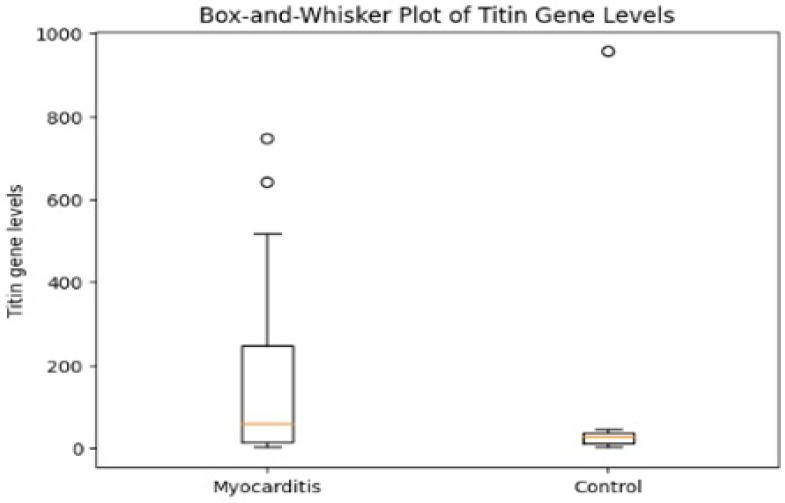
Titin expression shows greater variability and higher overall levels in the myocarditis group compared with controls.

PBMCs analysis revealed significantly increased *TTN* gene expression in PBMCs from myocarditis patients compared with controls (*p* = 0.015), corresponding to a 2.8-fold median increase, as illustrated in Figure 1 (box-and-whisker plot).

**Figure 2:**
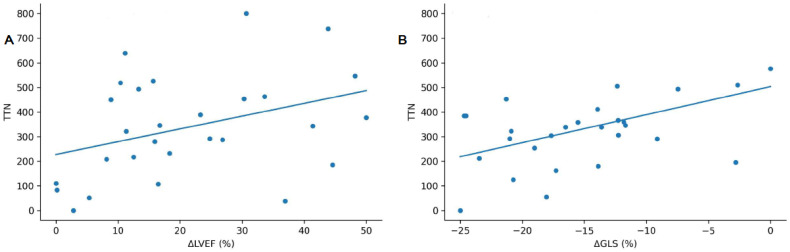
Correlation of titin (*TTN*) gene expression levels in PBMCs with (**A**) the changes in left ventricular ejection fraction (ΔLVEF) and (**B**) global longitudinal strain (ΔGLS) (Spearman’s r = 0.576, *p* < 0.001).

**Figure 3:**
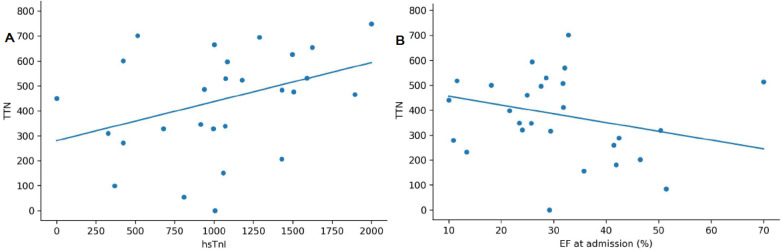
Correlation of titin (*TTN*) gene expression levels in peripheral blood mononuclear cells with (**A**) peak high-sensitivity troponin I levels (hsTnI) and (**B**) left ventricular ejection fraction (EF) at admission.

TTN expression demonstrated robust associations with markers of myocardial injury and systolic dysfunction. Specifically, TTN expression showed a moderate inverse correlation with baseline LVEF (r = −0.421, *p* = 0.025), a positive correlation with the magnitude of LVEF recovery (ΔLVEF) (r = 0.413, *p* = 0.029) (Figure 2), a strong positive correlation with GLS impairment (Spearman’s r = 0.576, *p* < 0.001), and a moderate positive correlation with peak hs-cTnI levels (r = 0.435, *p* = 0.021; (Figure 3).

During follow-up, myocarditis patients demonstrated significant functional recovery, with a median ΔLVEF of +2.5% (interquartile range [IQR] 0–10.0%; *p* = 0.012) and a ΔGLS of −3.0% (IQR −4.0 to −1.0%; *p* < 0.001) compared with baseline. Despite this improvement, GLS remained numerically lower than in controls [−20.0% (−21.0 to −19.0) vs. −21.0% (−21.75 to −19.25), *p* = 0.202], suggesting persistent subclinical systolic dysfunction.

Multivariable linear regression analyses confirmed TTN expression as an independent predictor of systolic function. After adjustment for hs-cTnI, TTN expression remained independently associated with baseline LVEF (unstandardized B = −0.011, standardized β = −0.225, *p* = 0.034; 95% CI −0.021 to −0.001). Moreover, TTN expression independently predicted LVEF recovery (ΔLVEF) (B = 0.009, β = 0.212, *p* = 0.050; 95% CI 0.000 to 0.019), supporting its prognostic value beyond traditional myocardial injury markers.

Collectively, these findings identify circulating PBMC TTN overexpression as a novel biomarker in acute myocarditis, reflecting the severity of myocardial injury, correlating with subclinical systolic dysfunction, and independently predicting functional recovery of left ventricular ejection fraction.

## 4. Discussion

This is the first study examining *TTN* gene expression in PBMCs in acute myocarditis. Our findings reveal that *TTN* gene expression levels are increased in peripheral blood monocytes of patients with acute myocarditis and may represent a novel biomarker linking peripheral immune activation to myocardial sarcomeric stress and providing mechanistic insight into disease severity and functional trajectory. PBMC *TTN* gene expression independently predicted both acute systolic impairment and long-term functional recovery beyond conventional biomarkers, establishing its incremental clinical value.

A fundamental clinical challenge in myocarditis is explaining why only a subset of individuals exposed to common triggers—predominantly viral infections—develop clinically significant myocardial inflammation, exhibiting highly variable phenotypes, from spontaneous resolution to fulminant heart failure or chronic dilated cardiomyopathy [20,21,22,23]. Host genetic susceptibility has emerged as the primary determinant, orchestrating interindividual differences in immune activation, viral clearance, and myocardial resilience to inflammatory injury [5,24,25].

It is well established that a maladaptive immune response to environmental triggers plays a central role in myocarditis pathophysiology. Genetic loci regulating immune responses to infection further support the concept that interindividual genetic variability influences susceptibility to myocardial inflammation, disease presentation, and clinical course. In parallel, growing evidence for genetic basis in other cardiovascular diseases—particularly cardiomyopathies—has led to the hypothesis that genetic heterogeneity may also contribute to the wide spectrum of clinical manifestations and outcomes observed in myocarditis [20].

Recent studies have focused on determining whether genetic variants and potentially pathogenic mutations influence the clinical evolution of myocarditis, favoring either complete recovery or progression to DCM [6]. These data support a model in which the clinical course of myocarditis reflects a complex interaction between genetic predisposition and the inflammatory insult. Proposed mechanisms include interactions between viral proteases and cytoskeletal components, ultimately predisposing affected individuals to persistent left ventricular dysfunction following the acute cellular inflammatory phase [6,7].

Supporting these observations, a recent study demonstrated that nearly 30% of patients with biopsy-confirmed myocarditis—particularly those presenting with heart failure and left ventricular systolic dysfunction—harbored pathogenic or likely pathogenic variants in cardiomyopathy-associated genes. Mutations in the *TTN* gene were the most frequently identified and were associated with a reduced likelihood of functional recovery over time [9]. TTN, a giant structural and signaling protein of striated muscle, can participate in intracellular signaling networks and cytoskeletal organization, potentially influencing cell activation, trafficking, and interactions with other tissues.

Investigating TTN expression in peripheral blood is supported by emerging evidence that titin is not restricted to cardiomyocytes, but is also expressed in endothelial cells, fibroblasts, and immune lineages, where it participates in cytoskeletal organization and mechanosensitive signaling. The detection of marked *TTN* gene overexpression specifically in PBMCs during acute myocarditis—represents the first documentation of sarcomeric protein dysregulation in circulating immune cells during human myocardial inflammation. This discovery challenges the traditional paradigm that titin signaling is confined to cardiomyocytes, revealing previously unrecognized syst emic dimensions of TTN biology. PBMCs, as primary myocardial infiltrators, likely serve as peripheral biosensors of sarcomeric stress, upregulating TTN mRNA in response to circulating danger signals [26,27].

In the human heart, TTN is expressed mainly as the stiff N2B and more compliant N2BA isoforms, whose ratio governs myocardial passive stiffness and diastolic function. Although we quantified total TTN mRNA in PBMCs rather than specific isoforms, our findings raise the hypothesis that inflammatory activation in acute myocarditis may be associated with myocardial TTN isoform remodeling, which should be addressed in future isoform-resolved myocardial studies.

TTN’s non-cardiomyocyte expression in endothelial cells, fibroblasts, and now immune lineages enables stiffness-dependent signaling via its kinase domain and binding partners (e.g., N2B element). In myocarditis, PBMC TTN upregulation may drive pathological stiffness-sensing, enhancing TGF-β secretion, matrix metalloproteinase activation, and profibrotic differentiation—explaining the strong correlations with persistent GLS impairment despite LVEF recovery [24,28,29]. However, in interpreting our results, it is important to emphasize that the proposed links between elevated PBMC TTN expression, immune activation, and myocardial fibrosis should be regarded as hypotheses rather than proven causal mechanisms. Our mechanistic framework is based on integration of existing experimental work on TTN biology and is intended to provide a biologically plausible context for the observed associations, not to imply direct proof of pathway involvement. Future studies combining isoform-resolved myocardial TTN assessment, detailed immune phenotyping, and functional experiments in immune cells will be required to validate or refute these hypotheses and to delineate the precise role of TTN in immune–cardiac cross-talk.

Notably, our cohort showed a marked male predominance, which is consistent with large contemporary registries and reviews reporting myocarditis to be more frequent in men than women, especially in adolescents and young adults [30].

Our findings demonstrate associations between PBMC TTN expression, the severity of systolic dysfunction at baseline, and functional recovery at one-year follow-up (ΔLVEF and GLS) but do not allow firm conclusions regarding long-term structural remodeling or clinical outcomes.

## 5. Limitations

While this study provides clear and robust evidence of the distinct PBMC TTN expression in acute myocarditis, several primary limitations must be acknowledged. The pilot nature with a modest sample size, although sufficient to detect strong main effects and multivariate relationships, precludes subgroup analyses by viral etiology, treatment regimens, or demographic factors, as well as formal adjudication of hard clinical endpoints such as heart failure hospitalization or mortality. Additional limitations include the absence of serial sampling to characterize TTN expression dynamics across the acute-to-chronic transition, and reliance on mRNA quantification without corresponding protein-level validation in PBMCs or myocardial tissue. The mechanistic causality linking PBMC TTN upregulation to myocardial sarcomeric injury remains correlative, requiring functional studies such as viral protease inhibition assays or TTN knockdown models. There was a marked sex imbalance in our cohort, with a predominance of male patients with myocarditis; given the small sample size, sex-adjusted or sex-stratified analyses were not feasible and potential sex-related differences could not be formally explored. Another key limitation is that we quantified TTN at the mRNA level only. Protein-level validation in PBMCs and, where feasible, myocardial tissue using techniques such as Western blotting or ELISA will be essential to confirm that transcriptional upregulation translates into altered titin protein abundance and to define the most feasible assay platform for clinical implementation

In our study, we did not perform additional separation or counting of purified monocytes; TTN mRNA expression was measured in total PBMC preparations. PBMCs, comprising lymphocytes and monocytes, were used as the source material; no additional purification step to isolate a pure monocyte fraction was performed, so TTN mRNA expression reflects its abundance within the mixed PBMC population, where monocytes typically account for approximately 10–30% of cells.

We did not adjust for additional covariates such as age, sex, or inflammatory markers, as inclusion of multiple predictors in this pilot cohort would have substantially increased the likelihood of overfitting. Finally, generalizability beyond presumed viral myocarditis phenotypes—our cohort’s predominant etiology—to immune-mediated, toxic, or idiopathic forms remains untested. Despite these constraints, the consistent, statistically robust findings across multiple complementary endpoints (injury markers, systolic deformation, and functional recovery) establish a compelling foundation for larger multicenter validation studies. Viral myocarditis is the most frequent clinical phenotype, but immune-mediated, toxic, or idiopathic forms show distinct immune signatures and may exhibit different PBMC transcriptional responses. Therefore, our findings on PBMC TTN overexpression should not be extrapolated to non-viral myocarditis without caution, and dedicated studies in autoimmune, drug-induced, and idiopathic myocarditis are warranted.

## 6. Conclusions

Our study demonstrates robust TTN elevation in patients’ PBMCs compared to healthy controls, with strong correlations to established injury markers and sensitive measures of subclinical systolic dysfunction. TTN dysregulation links genetic predisposition and myocardial injury, positioning PBMC TTN as a biomarker and therapeutic target. However, beyond identifying PBMC TTN mRNA as a promising biomarker of acute systolic impairment and early functional recovery in myocarditis, our findings also raise important mechanistic questions about the role of titin in circulating immune cells and why their transcriptional response appears to mirror cardiomyocyte stress, which should be addressed in future experimental studies.

## Figures and Tables

**Table 1:** Biochemical and echocardiographic parameters in the control and myocarditis groups.

	Control Group (*n* = 12)	Myocarditis Group (*n* = 29)	*p*
White blood cells (cells/μL)	6700 (5100–8500)	14,700 (10,100–18,500)	<0.001
C-reactive protein (mg/dL)	1.1 (0.4–2)	7.5 (6.4–18.2)	<0.001
hs-Troponin I (ng/L)	0.20 (0.10–0.30)	4250.0 (2757.0–6.575.0)	<0.001
*TTN* gene expression levels	21.47 (12.36–28.57)	59.15 (11.50–323.19)	0.015
GLS at baseline (%)	−20.33 ± 1.72	−16.62 ± 2.60	<0.001
GLS at one-year follow-up (%)	−21.0 (−21.75– 19.25)	−20.0 (−21–−19.0)	0.202
Left ventricular end-diastolic diameter (mm)	45 (43–52)	49 (45–58)	0.07
Right ventricular basal diameter (mm)	36 (33–37)	38 (34–43)	0.4
LVEF at baseline (%)	60.0 (60.0–60.0)	55.0 (50.0–60.0)	0.016
LVEF at one-year follow-up (%)	60.0 (60.0–60.0)	60.0 (60.0–60.0)	0.896
ΔGLS (%)	0 (−0.75–0.75)	−3.0 (−4.0–−1.0)	<0.001
ΔLVEF (%)	0 (0–0)	2.5 (0–10.0)	0.012

GLS: global longitudinal strain; hs-Troponin I: high-sensitivity troponin Imn; LVEF: left ventricular ejection fraction; TTN: titin. *p* < 0.005 is statistically significant.

## Data Availability

The original data presented in this study are openly available on Figshare at https://doi.org/10.6084/m9.figshare.12345678.

## Abbreviations

The following abbreviations are used in this manuscript:

MRI	Magnetic Resonance Imaging
GLS	Global Longitudinal Strain
hs-Troponin I	High-Sensitivity Troponin I
LGE	Late Gadolinium Enhancement
LVEF	Left Ventricular Ejection Fraction
TTN	Titin
